# A study of cognition and functional outcome in patients acutely admitted to hospital with schizophrenia, mania or depression

**DOI:** 10.1192/j.eurpsy.2026.10533

**Published:** 2026-07-31

**Authors:** G. Sachs, A. Erfurth

**Affiliations:** 1 Medical University of Vienna; 2 1st Department of Psychiatry and Psychotherapeutic Medicine, Klinik Hietzing, Vienna, Austria; 3 University of Münster, Münster, Germany

## Abstract

**Introduction:**

Achieving a favourable functional outcome is significantly influenced by neurocognition. A previous pilot study investigated whether cognitive dysfunction improves during inpatient treatment, in which acutely admitted patients receive a combination of pharmacological treatment and cognitive remediation (Maihofer et al. J Clin Med 2024, 13, 4843).

**Objectives:**

The present study aims to investigate the extent to which the functional outcome of patients diagnosed with psychotic and affective disorders is associated with cognitive function over time.

**Methods:**

This ongoing study employed the Screen for Cognitive Impairment in Psychiatry (SCIP-G: Sachs et al. Schiz Res Cogn 2021, 25, 100197; Sachs et al. Schiz Res Cogn 2022, 29, 100259) to evaluate 311 psychiatric inpatients aged 18–66 years (181 female, 130 male). The International Classification of Diseases, Tenth Revision (ICD-10) research criteria were used to determine patient diagnoses in this study. 105 patients were diagnosed with F2 (schizophrenia, schizoaffective and delusional disorders), 40 patients met the criteria for bipolar mania, 39 for bipolar depression and 127 patients met the criteria for unipolar depression (F32/F33). All patients received state-of-the-art pharmacotherapy and cognitive remediation using the COGPACK® software package version 6.06. Functioning was assessed using the Global Assessment of Function (GAF).

**Results:**

A significant difference was found in SCIP scores between time points 1 and 2 (F = 175.8, p < .001). Furthermore, significant improvement was observed in all four diagnostic groups (p < .001). There was no significant interaction between time and diagnosis, indicating that cognitive abilities improved significantly in all groups, with similar trends observed over time. A significant interaction effect was found between SCIP and GAF scores at both time points (p < .044). This is consistent with the idea that better cognitive performance is associated with a better functional outcome. No significant effect of diagnosis group was found (GAF/SCIP x diagnosis, p = .193).

**Conclusions:**

Cognitive dysfunction is commonly found in people with psychiatric disorders and can easily be assessed during an inpatient hospital stay with the SCIP. Treatment in an inpatient setting, which includes pharmacotherapy and cognitive training, improves cognitive deficits and overall functioning. Improvement in cognitive performance is observed to a similarly high degree in patients with psychotic disorders, bipolar disorder and depression.

**Disclosure of Interest:**

None Declared

